# A Boy’s Temper

**Published:** 1890-08

**Authors:** 


### A BOY'S TEMPER.

A boy is none the worse for possessing a little swagger and self-assertiveness, and any attempt on the part of parents to break his temper is a step in the wrong direction. It was said of the mother of the Wesleys that she tried to crush the self-will of her son John, yet any one who knows any thing of the history of John Wesley, is ready to admit that he was one of the most stubborn of men when once his opinion was formed upon a subject. Properly developed, a boy's self-confidence may blossom into a noble decision of character, which will be of infinite service to him in the struggle of life. In these days of keen competition, when every year makes the world's prizes harder to get, the victories and rewards of life are for those who, by indomitable pluck and energy, are able to grasp and retain their own. The child who grows to manhood with a broken spirit, must inevitably go to the wall. I do not say that a boy must be allowed all his own way, and have the privilege of riding rough-shod over the household rules and usages, or that his tendency to overbearing and rudeness should be encouraged. By all means, give him a taste of the birch, when the power of persuasion fails to bring him to reason. Let him know that he is under government ; that he has a place in the home where he will find a welcome and shelter as long as the old nest remains ; that his boyish exuberances and frolicsomeness will, to an extent, be tolerated; but so far and no further must he go without experiencing the weight of parental displeasure. The boy who has a fair share of elbow room for himself, yet knows that there are certain well defined bounds over which he must not pass, is almost certain to thrive both in physique and character. But the one who is constantly lectured for little offences, whipped when he hardly knows why, and in various ways reminded of his own unworthiness, will cherish a dislike for his childhood's home, and will leave it without a pang of regret.—*Farm and Home. Eng.*

---

A Reliable Safe.—We advertise the safes of two makers, that of the Victor Safe and Lock Co., Cincinnati, and E. C. Morris & Co., Boston. These safes are being sold in large numbers here and elsewhere, and give complete satisfaction, especially after a destructive fire, when their contents are found unscathed.

To Kill Roaches.—The following recipe from the *Druggists' Circular* is said to make a powder effective in driving away roaches. The preparation is said to be practically identical with "Peterman's Roach Food :" Borax, 37 oz., starch, 9 oz., cocoa, 4 oz. Thoroughly mix, and leave the unbidden guests to help themselves.

